# Perfect chronic skeletal muscle regeneration in adult spiny mice, *Acomys cahirinus*

**DOI:** 10.1038/s41598-018-27178-7

**Published:** 2018-06-11

**Authors:** Malcolm Maden, Jason Orr Brant, Andres Rubiano, Aaron Gabriel W. Sandoval, Chelsey Simmons, Robert Mitchell, Henry Collin-Hooper, Jason Jacobson, Saleh Omairi, Ketan Patel

**Affiliations:** 1 Department of Biology & UF Genetics Institute, University of Florida, Florida, USA; 2 Department of Aerospace & Mechanical Engineering, University of Florida, Florida, USA; 3 School of Biological Sciences, University of Reading, Reading, England; 4 College of Medicine, Wasit University, Kut, Iraq

## Abstract

The spiny mouse, *Acomys cahirinus*, is an adult mammal capable of remarkable feats of scar-free tissue regeneration after damage to several organs including the skin and the heart. Here we investigate the regenerative properties of the skeletal muscle of *A*. *cahirinus* tibialis anterior in comparison to the lab mouse, *Mus musculus*. The *A*. *cahirinus* TA showed a similar distribution of myosin heavy chain fibre types and a reduced proportion of oxidative fibres compared to *M*. *musculus*. There were differences in the matrix components of the TA with regard to collagen VI and the biomechanical properties. *A*. *cahirinus* TA regenerated faster with a more rapid induction of embryonic myosin and higher levels of dystrophin than in *M*. *musculus* fibres. There were lower levels of inflammation (NF-kB), fibrosis (TGFβ-1, collagens) and higher levels of the anti-inflammatory cytokine Cxcl12. There was a difference in macrophage profile between the two species. After multiple rounds of muscle regeneration the *M*. *musculus* TA failed to regenerate muscle fibres and instead produced a large numbers of adipocytes whereas the *A*. *cahirinus* TA regenerated perfectly. This clearly improved regeneration performance can be explained by differing levels of growth factors such as adiponectin between the two species.

## Introduction

Skeletal muscle is one of the few mammalian tissues which can rapidly repair itself repeatedly throughout life^1,2^. Following physical or toxic insults there is a rapid breakdown of the sarcolemma and an infiltration of phagocytic neutrophils and macrophages which ingest the degenerating muscle cell cytoplasm. In mice, neutrophil numbers peak first and then macrophage numbers rise over the first 3–4 days after damage as they phagocytose the muscle cell debris, but macrophage numbers continue to remain high as they subsequently become associated with proliferating satellite cells and newly regenerated muscle fibres where they play a positive role in myogenic cell differentiation and myofibre formation^3–5^. Reduced macrophage numbers results in delayed regeneration, reduced fibre size and an increase in adipocyte numbers^6–9^.

Satellite cells, located between the basal lamina and the sarcolemma of the myofibre, are the muscle stem cells and provide the source of new cells for regenerated muscle fibres. When they are genetically deleted no muscle regeneration occurs^10,11^. Satellite cells exist in a quiescent state in uninjured muscle and upon injury they are activated becoming muscle precursors which either return to their quiescent state or go on to differentiate into myoblasts. The latter then fuse with damaged fibres or each other to generate new fibres which are of small caliber with centrally located nuclei. There is a highly coordinated sequence of gene expression patterns during regeneration including the re-expression of embryonic and developmental myosins and myogenic regulatory factors such as embryonic myosin heavy chain, MyoD, myf5 and myogenin to ensure the appropriate fully differentiated product^1,12^.

Satellite cells are maintained in their niche by their unique gene expression patterns such the balance between Notch and Wnt signaling^13–15^ the balance between FGF/sprouty signaling^16^ and also by paracrine factors^17,18^ and components of the extracellular microenvironment such as collagen VI, fibronectin and tenascin-C^19–21^. Not only is the nature of the niche maintained by these extracellular molecules but it is also regulated by their biomechanical properties^22^ which controls physiological stiffness, tissue elasticity and the differentiative properties of stem cells^23^. Elaborating this balance of intrinsic, extrinsic factors and biomechanical properties is not only crucial for understanding the decline of regenerative ability with age but also for understanding the role that the connective tissue matrix plays in normal regeneration.

A novel cell type involved in the regeneration of myofibres has recently been described. This is the fibro/adipogenic progenitor (FAP), a bipotential cell which can generate either fibroblasts or adipocytes after chronic injury^24,25^. During normal regeneration FAPs are stimulated to enter the cell cycle and provide a source of growth factors such as IL-6 which increase myoblast differentiation. The fate of the FAPs is controlled by IL-4/IL-13 signaling from eosinophils which inhibits their spontaneous differentiation into adipocytes and instead permits them to generate growth factors for myoblast proliferation and differentiation^26^. This cell type is therefore at the heart of the process of fatty degeneration of skeletal muscle which occurs in myopathies such as Duchenne muscular dystrophy^27^.

In the work reported here we have examined the properties of skeletal muscle and its regeneration in the spiny mouse, *A*. *cahirinus*. This remarkable mammal has previously been shown to be capable of completely regenerating skin wounds in a scar-free manner and thus regenerating hairs, smooth muscle of the erector pili muscles, sebaceous glands, the skeletal muscle of the panniculus carnosus, adipose cells and the dermis^28–30^. Importantly, during this regenerative process the hole in the panniculus carnosus muscle regenerates in the absence of the connective tissue matrix, equivalent to volumetric muscle loss which cannot be recovered from in other mammals. *A*. *cahirinus* can also regenerate holes punched through the ear, thereby regenerating all the tissues of the skin plus the cartilage^28,31^. In addition, after a myocardial infarction there is very little fibrotic scarring and a full restoration of cardiac function. In view of the striking ability of *A*. *cahirinus* to regenerate all three types of muscle we were interested in comparing *A*. *cahirinus* and *M*. *musculus* in the standard skeletal muscle regeneration model, the myotoxin-damaged tibialis anterior (TA). We find that *A*. *cahirinus* TA can regenerate slightly faster than *M*. *musculus* but the major differences in the two species lie in the macrophage profiles which rapidly infiltrate the damaged muscle, the matrix and biomechanical properties of the muscle and the fact that repeated rounds of regeneration results in degenerated muscles with significant adipocyte infiltration in *M*. *musculus*, but perfect regeneration in *A*. *cahirinus*. We discuss potential causes of this improved regenerative ability in this novel mammalian model of regeneration.

## Results

### Comparison of the normal *M*. *musculu*s and *A*. *cahirinus* muscle

**Figure 1:**
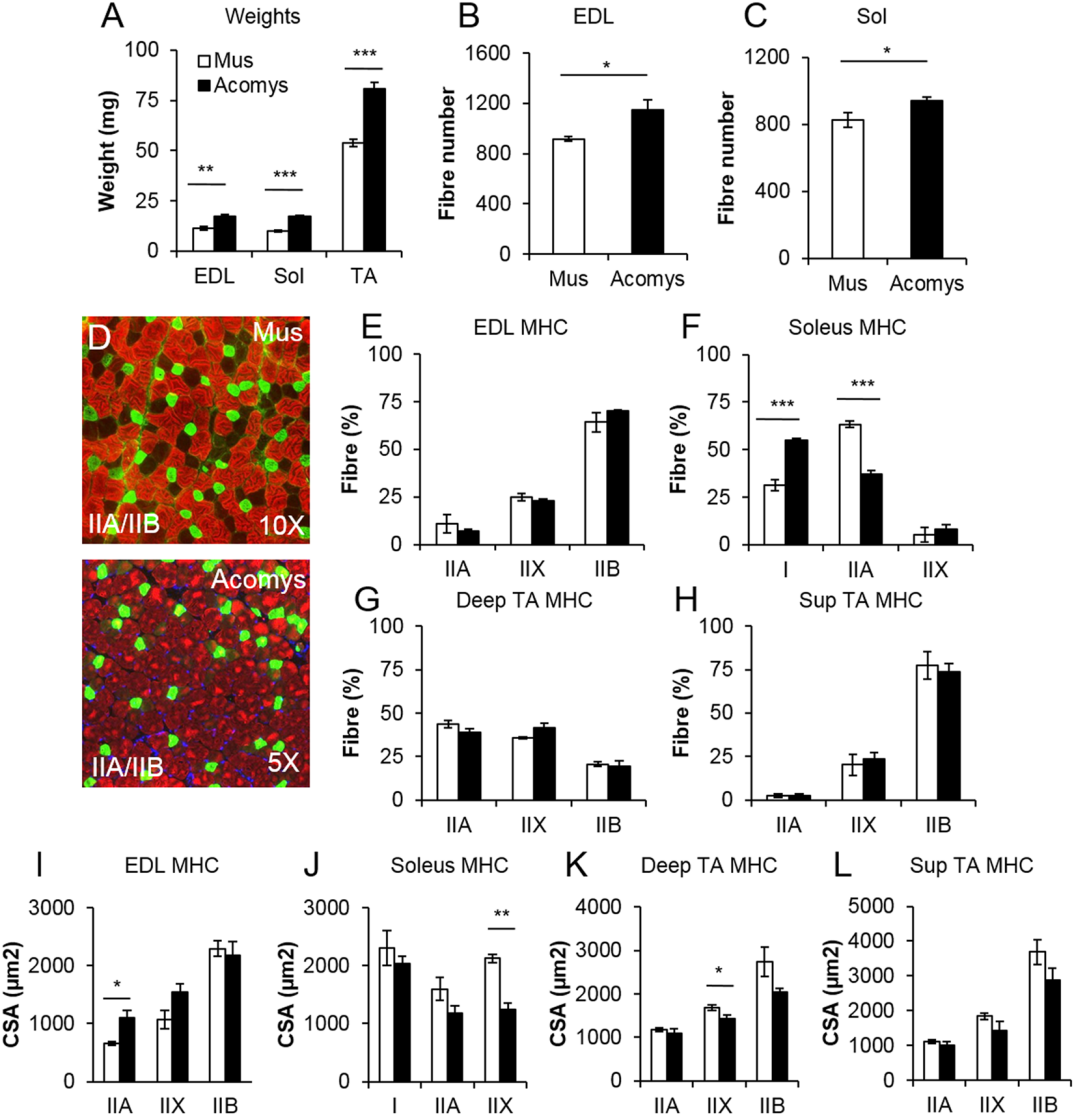
(**A**) Cahirinus skeletal muscle mass increase through fibre hyperplasia. (**A)** Skeletal muscle mass in *A*. *cahirinus* compared to C57/Bl6. (**B**,**C**) Fibre number increase in EDL and soleus of *A*. *cahirinus* compared to C57/Bl6. (**D**) Example of MHCIIA and MHCIIB protein expression in the EDL muscle from *M*. *musculus* and *A*. *cahirinus*. (**E**–**H**) MHC fibre distribution in (**E**) EDL, (**F**) Soleus, (**G**) deep TA and (**H**) superficial TA from *M*. *musculus* and *A*. *cahirinus*. (**I**–**L**) Comparison of cross-sectional fibre areas assigned to specific MHC isoforms for the (**I**) EDL, (**J**) Soleus, (**K**) deep TA and **(L)** superficial TA from *M*. *musculus* and *A*. *cahirinus*. n = 4/5 animals for each measure. Statistical analysis performed by two-tailed t-test. ^*^<0.05, ^**^<0.01, ^***^p < 0.001.

**Figure 2:**
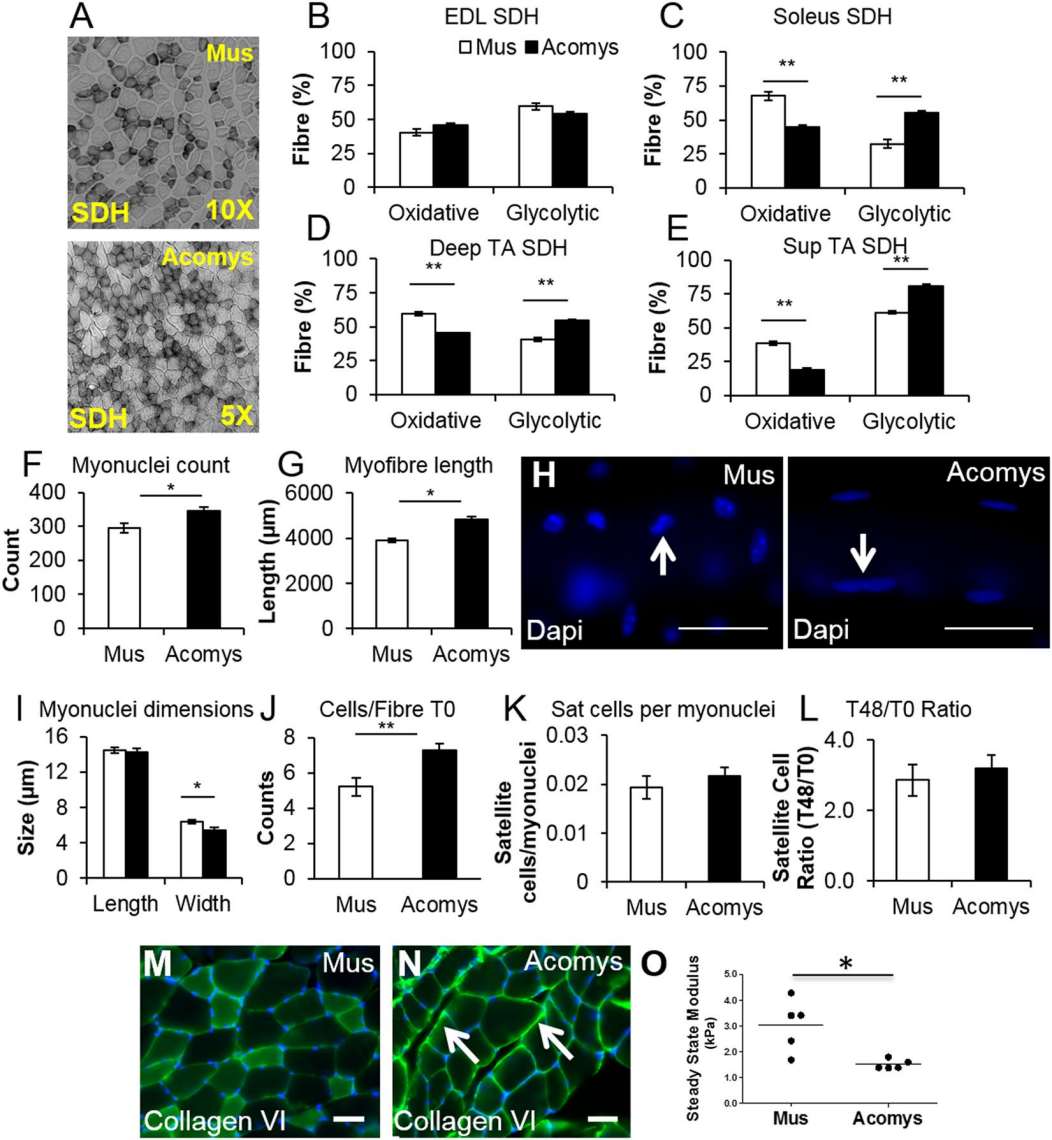
Metabolic, connective tissue and stiffness of *A*. *cahirinus* skeletal muscle and satellite cell profiling. (**A**) SDH staining of EDL muscle from *M*. *musculus* and *A*. *cahirinus*. (**B**–**E**) SDH activity in (**B**) EDL, (**C**) Soleus, (**D**) deep TA and (**E**) superficial TA from C57/Bl6 and *A*. *cahirinus*. Soleus and all parts of the TA display decreased proportion of SDH^+^ fibres compared to *Mus*. (**F**) Quantification of myonuclei content in fibres from the EDL of *M*. *musculus* and *A*. *cahirinus*. (**G**) EDL Myofibre length *M*. *musculus* and *A*. *cahirinus*. (**H**) DAPI stained nuclei in EDL fibres (arrow) of *M*. *musculus* and *A*. *cahirinus*. Scale bar = 150 μm. (**I**) Quantification of EDL myonuclei dimensions. (**J**) Quantification of satellite cell number on freshly isolated EDL fibres through Pax7 immunocytochemistry. (**K**) Frequency of EDL satellite cells relative to myonuclei. (**L**) Proliferation of satellite cells measured as a ratio of number of progenitors at 48 h in relation to the number of satellite cells at start of culture (T0). (**M**) Collagen VI immunofluorescence of normal EDL of *M*. *musculus* showing a decrease in immunoreactivity. Scale bar = 50 μm. (**N**) Collagen VI immunofluorescence of normal EDL of *A*. *cahirinus* showing strong immunoreactivity (arrows). Scale bar = 50 μm. (**O**) Measurements of the stiffness of freshly dissected TA muscle from *M*. *musculus* and *A*. *cahirinus*. Line shows the average value. Statistical analysis performed by two-tailed t-test. ^*^<0.05, ^**^<0.01.

Before examining any potential regenerative differences between *M*. *musculus* and *A*. *cahirinus* skeletal muscle we first determined whether there were any anatomical or physiological differences in C57Bl6 mice compared to *A*. *cahirinus*. We found that all hind limb muscles examined from *A*. *cahirinus* were heavier than their C57Bl6 counterparts (Fig. 1A) and both the EDL and Soleus from *A*. *cahirinus* contained significantly more fibres than their C57Bl6 counterparts (Fig. 1B,C). We next profiled the fibre composition and related this to their size to determine whether the enlargement of one particular fibre type was a factor in the increased muscle mass. Myosin heavy chain profiling showed that the muscle composition based on this criteria was similar in both species (Fig. 1D–H). Additionally there was no significant trend indicating a change in fibre size between the two species based on MHC categorization (Fig. 1I–L). Next we performed a biochemical profiling of *A*. *cahirinus* muscle to develop an understanding of the metabolic processes that supports its function. To this end we profiled the muscle for succinate dehydrogenase (SDH) activity and found that the *A*. *cahirinus* EDL has a similar SDH profile to its C57Bl6 counterpart whereas both the Soleus and TA (deep and superficial) had a greater proportion of SDH positive fibres than those of C57Bl6 mice (Fig. 2A–E). Next we examined individual muscle fibres from *A*. *cahirinus* and found significant differences in terms of their myonuclei and satellite cells in comparison to C57Bl6. EDL muscle fibres from *A*. *cahirinus* contained more myonuclei and were longer than their C57Bl6 counterparts (Fig. 2F,G). We also noted a difference in the shape of myonuclei between the two rodent species; those of *A*. *cahirinus* were significantly flatter compared to ones from C57Bl6 muscle (Fig. 2H,I). Next we profiled *A*. *cahirinus* satellite cells through the expression of Pax7 and found more on undamaged fibres compared to their C57Bl6 counterparts (Fig. 2J and Supplementary Fig. 1A–D). However when this measure was normalized to the change in myonuclei number per fibre, we found that there was the same number of satellite cells relative to myonuclei in both species (Fig. 2K). Thereafter we examined the proliferation rate of *A*. *cahirinus* satellite cells by culturing EDL fibres for 48 h, counting the number of progeny (Pax7^+^/MyoD^−^, Pax7^+^/MyoD^+^, Pax7^−^/MyoD^+^) and relating this to the number of satellite cells at the beginning of the experiment to generate a T48/T0 ratio. This approach revealed that satellite cells from the two species had identical proliferation characteristics (Fig. 2L). Importantly, all cells on the fibres at 48hrs were either Pax7^+^/MyoD^−^, Pax7^+^/MyoD^+^ or Pax7^−^/MyoD^+^. In summary, the hindlimb muscles from *A*. *cahirinus* were larger than their C57Bl6 counterparts due to an increase in myofibre number and length. Individual fibre size from *A*. *cahirinus* and C57Bl6 as well as MHC profile were similar, but the *A*. *cahirinus* TA had more oxidative fibres. Finally the number of satellite cells relative to myonuclei as well as satellite cell proliferation rate was identical in the two species.

### ECM components of normal TA muscle

**Figure 3:**
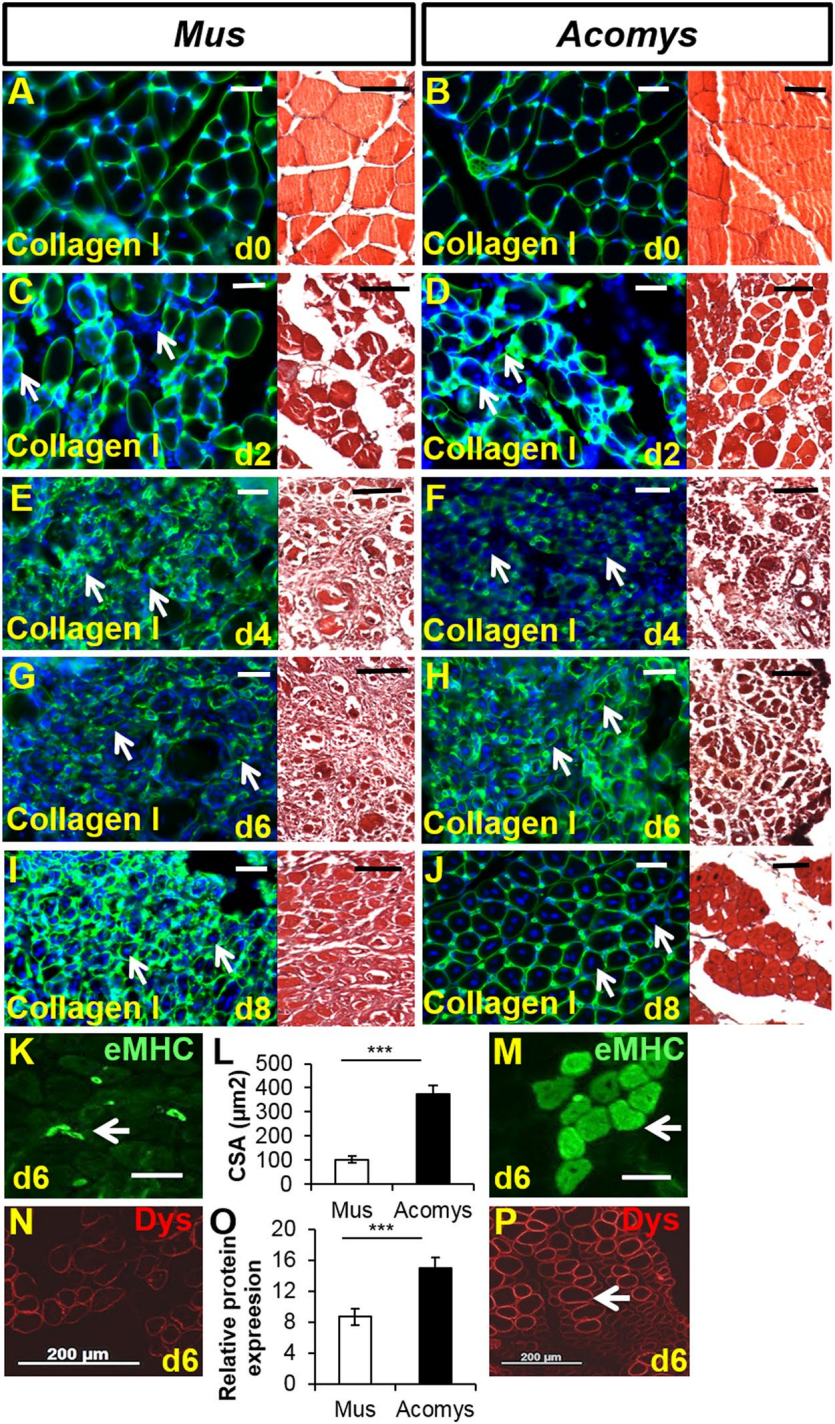
Dynamics of skeletal muscle regeneration after single CTX injection. Green = col I, blue = Hoescht stained nuclei. (**A**,**B**) Normal TA structure. Scale bars = 50 μm, same scales for **A**–**J**. (**C**,**D)** 2 days after CTX injection the myofibre membranes are losing their definition and many cells have invaded the area (arrows). (**E**,**F**) After 4 days there is complete destruction of the myofibres, a huge invasion of cells and proliferation of endogenous cells (arrows). (**G**,**H**) By day 6 there is a suggestion of reformation of myofibre membranes (arrows). (**I**,**J**) By day 8 new fibres have regenerated in *A*. *cahirinus* (right columns) with centrally located nuclei whereas in *M*. *musculus* (left columns) the process is by no means complete (arrows). (**K**–**M**) Examination of eMHC as a gauge of TA regeneration at day 6. (**K**) Example of eMHC^+^ fibres (arrow) in *M*. *musculus* and (**M**) *A*. *cahirinus*. (**L**) Quantification of regenerated fibres (eMHC^+^ fibre size). (**N**–**P)** Immunocytochemical staining and quantification for Dystrophin in regenerating areas of (**N**) *M*. *musculus* and (**P**) *A*. *cahirinus*. Note higher levels of Dystrophin in *A*. *cahirinus* (arrow). (**O**) Quantification of Dystrophin expression at the sarcolemma. Statistical analysis performed by two-tailed t-test. ^***^p < 0.001.

Although the muscle MHC fibre profiles were similar between *M*. *musculus* and *A*. *cahirinus* TAs we next considered whether there were any differences that could be detected in the ECM components of this muscle because of the role that the ECM plays in maintaining the stem cell niche^20–22^. Normal TAs were examined for the presence of a range of collagens (I, IV, VI, XII, XVII), fibronectin, laminin and tenascin C by immunofluorescence. The majority of ECM components showed similar levels of fluorescence between *M*. *musculus* and *A*. *cahirinus* (e.g. Fig. 3A,B, collagen I) except with regard to collagen VI which was significantly more immunoreactive in *A*. *cahirinus* than in *M*. *musculus* (Fig. 2M,N). There was very little immunoreactivity in normal sections of *M*. *musculus* TA (Fig. 2M), but strong reactivity in section of *A*. *cahirinus* TA in the expected location in the endomysium around the myofibres and more intensely in perimysial spaces between fascicles (Fig. 2N).

### Muscle biomechanical stiffness

Since Col VI levels were higher in *A*. *cahirinus* and in view of the role that this molecule plays in regulating the stem cell niche via the muscle biomechanical properties^22,23^ we next examined the physiological stiffness of freshly isolated *M*. *musculus* and *A*. *cahirinus* TA. Using a custom indentation device to measure stiffness calculated in terms of steady state modulus (SSM) we found that the TA of *M*. *musculus* samples had a SSM of 3.05 ± 1.09 kPa which is significantly stiffer than the value for *A*. *cahirinus* (1.52 ± 0.34 kPa, Wilcoxon non-parametric comparison, p < 0.02) (Fig. 2O). Since proliferative and migratory properties of cells can be regulated by ECM stiffness^32^ this difference seen here may play a role in the improved regenerative properties of *A*. *cahirinus*.

### Regenerative properties of *M*. *musculus* and *A*. *cahirinus* muscle

The regenerative response of the TA to cardiotoxin was studied by sampling both C57Bl6 and CD-1 *M*. *musculus* compared to *A*. *cahirinus* on days 2, 4, 6, 8, 10 and 16. The progress of regeneration was examined on wax sections stained with Mason’s trichrome or hematoxylin and eosin and frozen sections stained with antibodies to collagen I, IV, XII and embryonic myosin. As expected, fibre breakdown and subsequent regeneration was rapid in both species but was faster in *A*. *cahirinus* as fully formed regenerated fibres appeared earlier. By 2 days after myotoxin there was a huge influx of cells into the damaged muscles, but despite this the tissues retained their overall structure as can be seen in Fig. 3C,D although the collagen I immunohistochemistry showed that the muscle fibres in both species were beginning to look less defined. By day 4 there were even more cells within the muscles and the fibres had by now clearly degenerated leaving almost none present in the myotoxin injected area (Fig. 3E,F). By day 6 the *M*. *musculus* TA was still densely packed with cells with few obvious signs of fibre regeneration. Those few fibres present looked like they did on day 4 and had not completely degenerated (Fig. 3G). However in the day 6 *A*. *cahirinus* samples (Fig. 3H) there were clear signs of regenerating fibres with centrally located nuclei and small cross sectional areas although the intervening areas were still packed with cells and debris. This was further evidenced by quantifying the size of fibres that express embryonic Myosin Heavy Chain (eMHC) at day 6; eMHC^+^ from *A*. *cahirinus* were 3.5 times larger than those of *M*. *musculus* (Fig. 3K–M). Additionally each regenerated fibre from *A*. *cahirinus* expressed more Dystrophin at the sarcolemma (Fig. 3N–P). By day 8 *A*. *cahirinus* had almost completed regeneration and the damaged area now had fibres all with centrally located nuclei (Fig. 3J). By contrast the *M*. *musculus* samples at day 8 were clearly regenerating but the endomysial spaces were still full of debris and cells (Fig. 3I) and *M*. *musculus* did not reach an equivalent regenerative stage until 2 or 3 days later than *A*. *cahirinus*.

### Comparative gene expression during TA regeneration

To determine whether there was a simple molecular explanation for the increased speed of regeneration in *A*. *cahirinus* we compared *M*. *musculus* and *A*. *cahirinus* TA regeneration in terms of the temporal expression of myogenic factors, inflammatory markers and several collagens by qPCR at days 4, 6 and 16 relative to day 0 levels.

#### Myogenic factors

**Figure 4:**
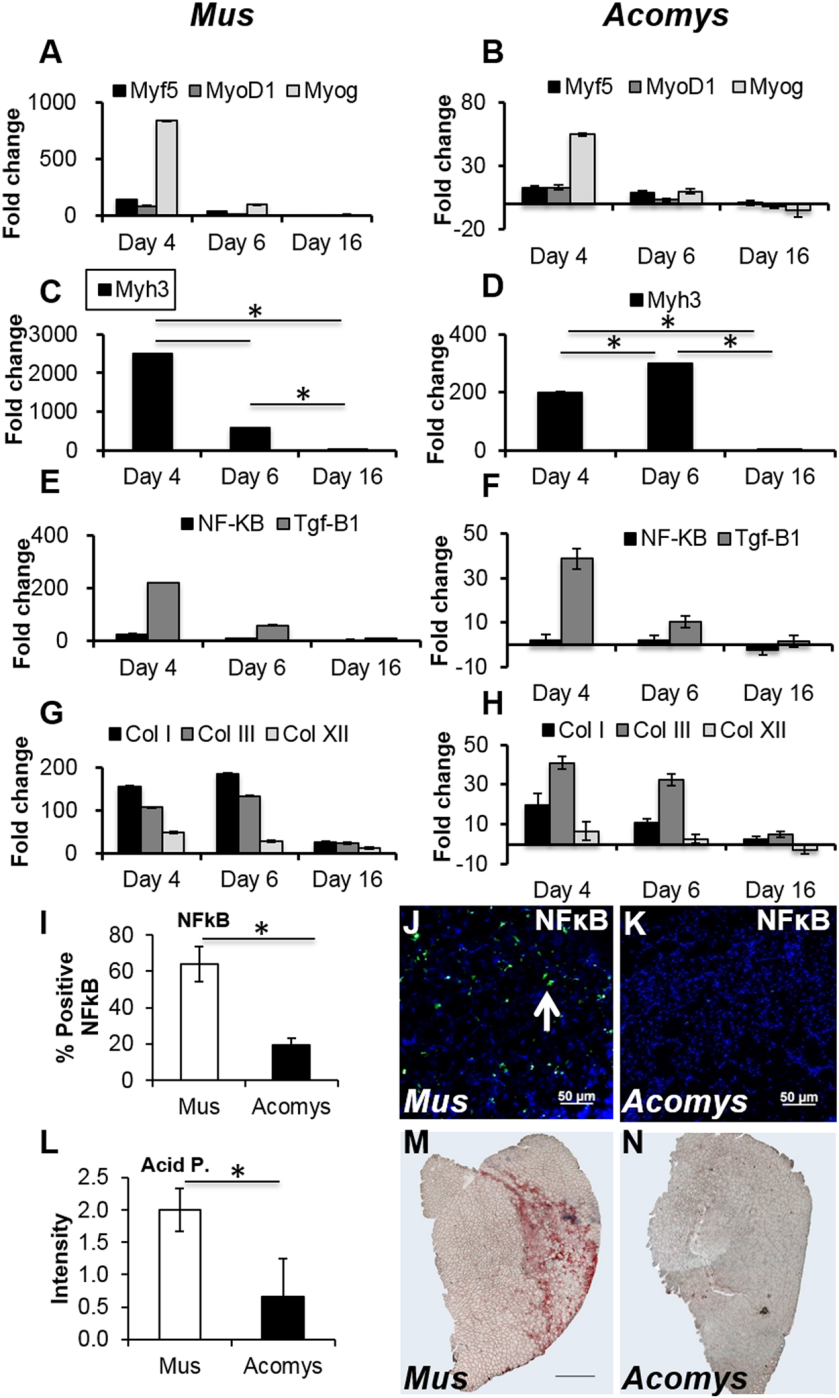
Gene expression profiling, NFκB and lysosomal activity of CTX damaged skeletal muscle. Days 4, 6 and 16 after CTX injection in *M*. *musculu*s (**A**,**C**,**E**,**G**) and *A*. *cahirinus* (**B**,**D**,**F**,**H**) TA. (**A–B)** Expression of *Myf5*, *MyoD1* and *Myogenin*. (**C**–**D**) Expression of *Myh3*. (**E**–**F**) Expression of NFκB. (**G**–**H**) Expression of Collagen *I*, *III* and *XII*. (**I**) Quantification of NFκB positive cells in regenerating TA at day 6. (**J**–**K**) Immunocytochemical images for NFκB expressing in regenerating TA at day 6 in *M*. *musculus* and *A*. *cahirinus* (white arrow). (**L**) Quantification of Acid Phosphatase activity in regenerating TA at day 6. (**M**–**N**) Histological stained images for Acid Phosphatase activity in regenerating TA at day 6 in *M*. *musculus* and *A*. *cahirinus*. Statistical analysis performed by two-tailed t-test. ^*^<0.05.

The expression levels of the myogenic factors *Myf5*, *MyoD1* and *Myog* peaked early, as expected on day 4 of regeneration in both species (Fig. 4A,B) and the expression levels of *Myog* was the highest of these three factors. The magnitude of the increase in these factors was very much higher in *M*. *musculus* than in *A*. *cahirinus*. With the embryonic myosin *Myh3*, again the magnitude of the increase was far greater in *M*. *musculus* than *A*. *cahirinus* (Fig. 4C,D). In *A*. *cahirinus* the increase in expression levels of *Mhy3* was more prolonged because it was still increasing on day 6 whereas in *M*. *musculus* on day 6 levels had dropped 5-fold compared to day 4.

#### Inflammation and fibrosis

Levels of *Nf-κB* showed the same temporal sequence in *M*. *musculus* and *A*. *cahirinus* (Fig. 4E,F) being present on both days 4 and 6 of regeneration. The magnitude of the increase compared to day 0 was higher in *M*. *musculus* than *A*. *cahirinus* suggesting there was relatively lower inflammation in *A*. *cahirinus*. *Tgf-*β*1* showed a similar pattern of temporal expression in both species peaking at day 4 (Fig. 4E,F) and as in the case of *Nf-κB* the magnitude of the increase compared to day 0 was far higher in *M*. *musculus* than in *A*. *cahirinus* suggesting that there was potentially less fibrosis in *A*. *cahirinus*.

The decreased levels of inflammation and fibrosis in *A*. *cahirinus* which this qPCR suggested were further verified in several ways. Firstly, examining regenerating muscle sections with immunocytochemistry using an antibody to NF-κB showed that the number of cells expressing NF-κB in regenerating muscle at day 6 was 3 times higher in *M*. *musculus* compared to *A*. *cahirinus* (Fig. 4I–K). NF-κB expression in Mus co-localised to the expression of the pan macrophage marker F4/80 (Supplementary Fig. 1E,F). Secondly, acid phosphatase activity, an indicator of muscle fibre necrosis, was virtually absent in *A*. *cahirinus* but clearly present in *M*. *musculus* (Fig. 4L–N). Thirdly, levels of fibrosis as measured by qPCRs for three collagens, *Col I*, *Col III* and *Col XII* levels showed far higher levels of increase compared to day 0 in *M*. *musculus* than in *A*. *cahirinus* (Fig. 4G,H). The expression of these genes in both species were highest on days 4 and 6 and then had dropped by day 16. In *M*. *musculus* col I levels were the highest of these three collagens, but in *A*. *cahirinus* the levels of col III was the highest.

**Figure 5:**
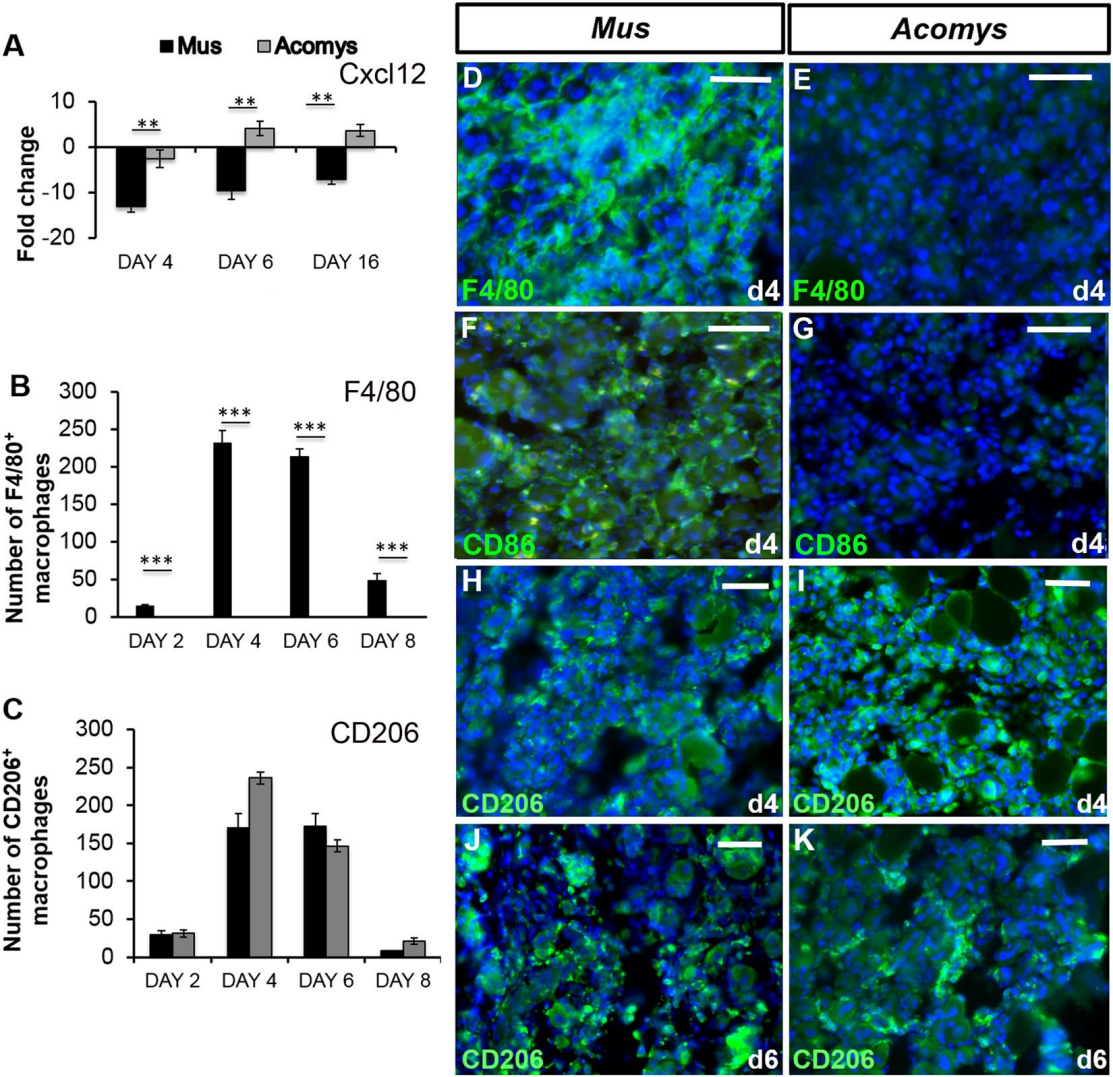
Macrophage profiling during muscle regeneration. (**A**) RT-qPCR analysis of gene expression of *Cxcl2* on days 4, 6 and 16 after CTX injection-induced injury. All results are relative to day 0 (normal TA) and error bars represent standard deviation. (**B**) counts of F4/80 macrophages showing a peak in *M*. *musculus* on days 4 and 6 and an absence in *A*. *cahirinus*. (**C**) counts of CD206 macrophages showing a peak at days 4 and 6 and equivalent numbers in *M*. *musculus* and *A*. *cahirinus*. (**D**–**E**) F4/80 immunocytochemistry in *M*. *musculus* (left column) and *A*. *cahirinus* (right column) in the TA at days 4 after CTX injection. Many F4/80 expressing cells (green) are seen in *M*. *musculus* whereas there is no positive reaction in *A*. *cahirinus*. Scale bars = 100 μm. (**F**–**G**) CD86 immunocytochemistry in *M*. *musculus* and *A*. *cahirinus* in the TA at days 4 after CTX injection. Many CD86 expressing cells (green) in *M*. *musculus* are seen whereas there is no positive reaction in *A*. *cahirinus*. Scale bars = 100 μm. (**H**–**K)** CD206 macrophage immunocytochemistry in *M*. *musculus* and *A*. *cahirinus* in the TA at days 4 and 6 after CTX injection. Both species show similar numbers of positive cells (green). Scale bars = 100 μm. Statistical analysis performed by two-tailed t-test. ^**^ < 0.01, ^***^p < 0.001.

Since we saw decreased levels of inflammation in the *A*. *cahirinus* TA during regeneration we looked at levels of *Cxcl12*, a chemokine that has been shown to have a novel anti-inflammatory role^33^, as well as a role in positively regulating muscle regeneration^34,35^. The change in expression of *Cxcl12* was greater in *A*. *cahirinus* compared to *Mus*, with a decrease in expression of −2.5x at day 4, a peak of expression at day 6 (4.1x) and still elevated levels (3.6x) at day 16. In *M*. *musculus* expression of *Cxcl12* was down-regulated at all time points, with expression −13x at day 4, −9.6x at day 6 and −7x at day 16 (Fig. 5A).

### Macrophage involvement in TA regeneration

We have previously observed an absence of the classically activated macrophage marker, F4/80, in *A*. *cahirinus* skin wounds during regeneration^30^ and the same is true of the regenerating ear^36^ so we next asked whether they were present during TA regeneration. Sections of both *M*. *musculus* and *A*. *cahirinus* muscle regeneration were examined over the first 8 days of regeneration when macrophages are at their highest levels for the presence of F4/80^+^ cells. *M*. *musculus* TAs showed a typical response with F4/80^+^ cells beginning to be detected at day 2, large numbers present at day 4 and day 6 (Fig. 5D) and then declining at day 8 of regeneration (Fig. 5B). In contrast there were no detectable F4/80 +ve cells in *A*. *cahirinus* regenerating TAs (Fig. 5B,E). Their presence in *Acomys* spleen^30^ (Supplementary Fig. 1G,H) and that their numbers in the bone marrow can be stimulated by IFNγ and LPS^36^ suggests this is not due to lack of antibody reactivity. In confirmation we examined another M1 marker previously used in *A*. *cahirinus*, the CD86 antibody^36^ and here too we could not detect any immunoreactivity in damaged *A*. *cahirinus* muscle (Fig. 5G), but there were many positive cells in *M*. *musculus* muscle (Fig. 5F). M2 macrophages, however, were present in abundance in *A*. *cahirinus*. Using the CD206 antibody there were equal numbers of M2 macrophages detected in the sections of *A*. *cahirinus* TA regeneration (Fig. 5I,K) compared to *M*. *musculus* in being just detectable at day 2, high numbers at days 4 and 6 (Fig. 5H–K) and then declining by day 8 of regeneration (Fig. 5C).

### Behavior of FAPS during TA regeneration

**Figure 6:**
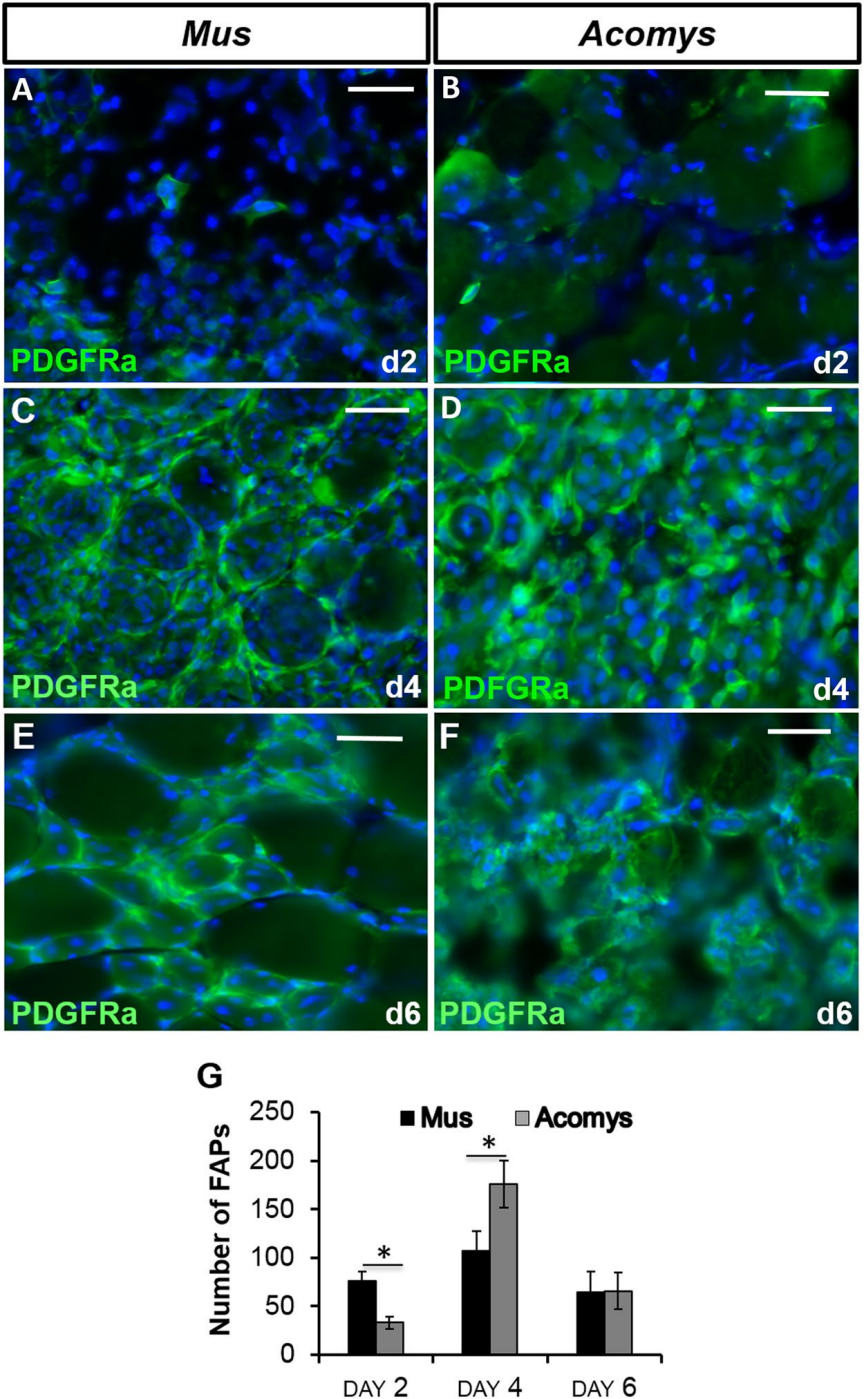
Fibroadipogenic cell profiling during skeletal muscle regeneration. (**A**–**F**) PDGFRα immunocytochemistry as a marker of FAPs in *M*. *musculus* (left column) and *A*. *cahirinus* (right column) TA at times indicated after CTX injection. Similar numbers are seen in both species at equivalent times. (**G**) Counts of PDGFRα+ FAPS in *M*. *musculus* and *A*. *cahirinus* showing peak levels at day 4. Statistical analysis performed by two-tailed t-test. ^*^<0.05.

We next looked at another cell type important for muscle regeneration, namely the FAP, identified by their expression of the platelet derived growth factor receptor alpha (PDGFRα). These cells were virtually undetectable in normal muscle, but appeared in large numbers in the endomysial spaces between the myofibres following CTX damage in both *M*. *musculus* and *A*. *cahirinus* (Fig. 6A–F). Cell counts revealed that their numbers peaked at day 4 of regeneration and there were more FAPs in *A*. *cahirinus* than in *M*. *musculus* (Fig. 6G).

### Multiple rounds of regeneration

Finally, we considered whether there may be ways of revealing more profound differences in the regenerative ability between these two species than just differences in timing. To this end we subjected the TAs to multiple rounds of regeneration. A group (n = 9) of *M*. *musculus* and *A*. *cahirinus* had five sequential myotoxin injections spaced 3 weeks apart and 3 weeks after the fifth injection the TAs were fixed for histology or prepared for RNA.

**Figure 7:**
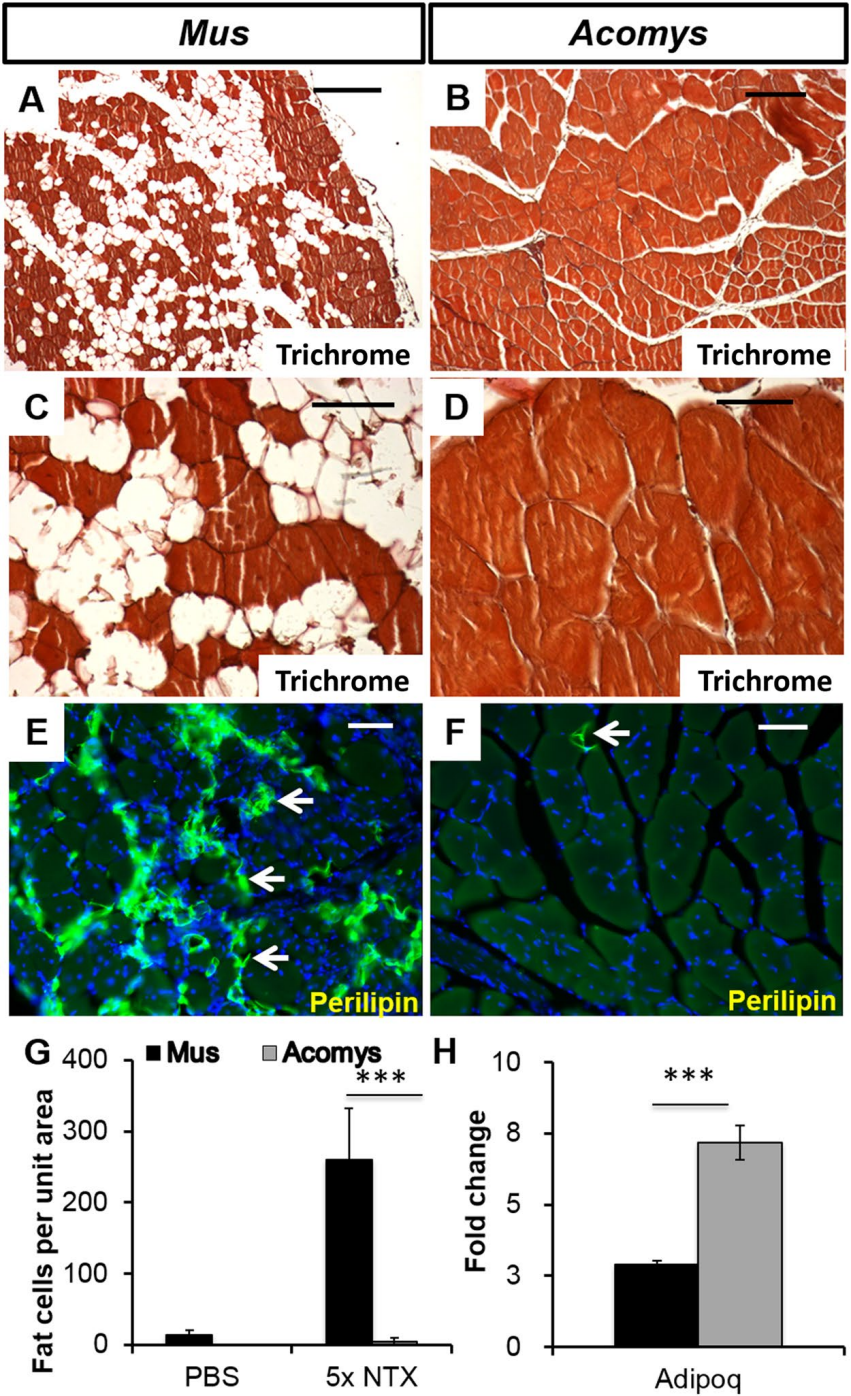
The effect of repeated rounds of cardiotoxin injury (5x) on muscle structure in *M*. *musculus* (left columns) and *A*. *cahirinus* (right columns). (**A**,**C**) Trichrome stained TA of *M*. *musculus* showing large gaps both within and between muscle fascicles. These are adipocytes (**E**). Scale bars = 100 μm (**A**) and 50 μm (**C**).(**B**,**D**) Trichrome stained TA of *A*. *cahirinus* showing perfect structural regeneration. Scale bars = 100 μm (**B**) and 50 μm (**D**). (**E**,**F**) Perilipin immunocytochemistry of *M*. *musculus* (**E**) showing many positive adipocytes (arrows) in the muscle compared to *A*. *cahirinus* (**F**) showing only one adipocyte present (arrow). Scale bars = 100 μm. (**G**) counts of adipocyte numbers in *M*. *musculus* and *A*. *cahirinus* TA after 5 rounds of repeated CTX injection into the TA showing almost no adipocytes present in *A*. *cahirinus*. (**H**) RT-qPCR analysis of gene expression for Adipoq after 5 rounds of CTX injections showing 2.5-fold higher levels in *A*. *cahirinus* (grey bar) which may contribute to its improved regenerative ability. Statistical analysis performed by two-tailed t-test. ^***^<0.001.

Control TAs subjected to 5 sequential injections of PBS showed excellent regeneration in both *M*. *musculus* and *A*. *cahirinus*. The only abnormality noted in histological sections of these controls was the presence of a very few fat cells in the perimysial connective tissue spaces. Cell counts revealed *M*. *musculus* control TAs averaged 13 adipocytes per section and in *A*. *cahirinus* there were 0 (Fig. 7G). In both these controls a few regenerated fibres with centrally located nuclei were present suggesting that 5 needle sticks causes some damage to muscle fibres and induces a degree of regeneration, but there was no obvious increase in fibrosis. After 5 myotoxin injections in *M*. *musculus*, however there was a dramatic decrease in the quality of regenerated TA and the histological sections showed the presence of large numbers of fat cells mostly in the perimysium but also scattered throughout the muscle fascicles (Fig. 7A,C). Counts of fat cell numbers in these sections revealed on average 260 fat cells per unit area (Fig. 7G). The presence of extensive adipocyte production was confirmed using immunocytochemistry with a perilipin antibody (Fig. 7E). In dramatic contrast there were almost no fat cells in *A*. *cahirinus* 5x CTX TAs and the histological sections showed perfect regeneration (Fig. 7B,D) with only an occasional cell stained for perilipin (Fig. 7F). Counts of these sections revealed an occasional fat cell giving an average value of 5 cells per unit area (Fig. 7G). This was not due to lack of reactivity of the antibody in *A*. *cahirinus* because the adipocytes in the hypodermis of the skin stain intensely with this antibody (Supplementary Fig. 2A). Furthermore, multiple rounds of regeneration led to a *M*. *musculus* muscle that was very poorly populated by satellite cells whereas their counterparts were still present in *A*. *cahirinus* (Supplementary Fig. 2B–E). However their low numbers precluded a meaningful quantification. Thus repeated rounds of regeneration results in perfect regeneration in *A*. *cahirinus* but a striking deposition of adipocytes in *M*. *musculus*. We lastly looked at the expression of adiponectin (*Adipoq*), a hormone that has recently been shown to be involved in skeletal muscle regeneration^37^. We found that *Adipoq* is 2.9 fold higher in *M*. *musculus* and 7.2 fold higher in *A*. *cahirinus* in 5x myotoxin tissues (Fig. 7H).

## Discussion

The spiny mouse, *A*. *cahirinus*, is a unique mammal in its ability to regenerate a remarkable array of tissues, notably scar-free regeneration of large skin excisional wounds and burns involving hairs, sebaceous glands, erector pili muscles, dermis^28–30^; ear punches involving these same skin components plus cartilage^28,31^ and can recover from myocardial infarction. In view of this ability to regenerate tissues which the typical mammal such as the lab mouse, *Mus*, or humans cannot it was of interest to examine the regeneration of a tissue which mammals *can* regenerate, namely skeletal muscle of the tibialis anterior after myotoxin injury to look for differences or improvements.

We first examined the anatomy of the normal TA and adjacent muscles of *M*. *musculus* and *A*. *cahirinus* which showed some differences. In *A*. *cahirinus* there were more and longer fibres with more myonuclei in them and the nuclei were of a flatter shape although there was no change in MHC fibre type. The *A*. *cahirinus* TA has a higher proportion of SDH positive fibres (more oxidative fibres) and higher numbers of satellite cells although when corrected for more myonuclei the relative abundance is the same and they proliferated to the same degree. The normal TA of *A*. *cahirinus* had a different muscle ECM composition, notably higher levels of collagen VI, a matrix component which plays a crucial role in satellite cell renewal and muscle regeneration^22^ and the regenerating TA of *A*. *cahirinus* had higher levels of dystrophin, a component of the dystrophin-glycoprotein complex which is vital for stabilizing muscle fibre membrane integrity^38^. The *col6a1*−/− mouse displays a depleted satellite cell pool, decreased numbers of regenerated myofibres and increased levels of fibrosis particularly after repeated rounds of myotoxin injury^22^ so differences in levels of this protein may contribute to regenerative differences between the two species.

Because of the role of the matrix and its biomechanical properties in stem cell differentiation^23^ we measured the elasticity of the TAs and determined that the effective stiffness of *M*. *musculus* with little col VI was 3.05 kPa and *A*. *cahirinus* with high col VI was 1.5 kPa. This is in the opposite direction to the *col6a1*−/− mouse where the muscles showed an increase in elasticity in its absence^22^, but our values for effective stiffness of murine skeletal muscle are within the range of previously reported direct characterization values (2–35 kPa)^39^. Reported indirect characterization values range from 7.6–128.5 kPa, though, which reinforces the difficulty of comparing characterization data across labs^40–43^. Nevertheless, there is surely a range of ECM molecules responsible for stiffness, not just collagen VI, another example being fibronectin^21^^.^. Using proteomics, tissue stiffness has been shown to be correlated to protein abundance, specifically varied levels of collagens in the extracellular matrix^44^. However, *mdx* mice have been used to show that collagen content is not predictive of muscle stiffness, even in collagen-rich, fibrotic skeletal muscle^45^. Regardless of the stiffness increase mechanisms, since proliferative and migratory properties of cells can be regulated by ECM stiffness^39^ and ECM proteins bind soluble growth factors that can affect many aspects of regeneration, tissue stiffness might help elucidate regenerative differences between *M*. *musculus* and *A*. *cahirinus*.

Following myotoxin injection into the TA there was a rapid breakdown of the myofibres in both *M*. *musculus* and *A*. *cahirinus* and a rapid regeneration of replacement fibres. *A*. *cahirinus* consistently regenerated faster than *M*. *musculus* in that fibre regeneration was histologically complete by around day 8 after damage whereas it took until around day 10 for *M*. *musculus* to complete the process. This increased speed was unlikely to be caused by differences in either satellite cell density or satellite cell proliferation between the two species as no differences could be seen in these two parameters. During the regenerative process eMHC expression appeared more prolonged and was present in larger fibres in *A*. *cahirinus* than in *M*. *musculus* although the absolute expression levels were higher in the latter. It seems that a large burst of eMHC expression followed by a rapid repression is characteristic of a slower regeneration rate and ultimately more error-prone regeneration in *M*. *musculus* than the more extensive but lower expression level seen in the *A*. *cahirinus* fibres.

There were also lower expression levels in *A*. *cahirinus* of the inflammatory and fibrosis genes we examined: *NF-*κ*B* as an inflammatory marker, *TGF-*β1 for fibrosis, several collagens and there were fewer cells positive by NF-κB immunocytochemistry and no cells showing necrosis by acid phosphatase activity. We do not believe that the muscle of *A*. *cahirinus* is more resistant to damage than *M*. *musculus* because both showed high levels of damage after CTX injection (Fig. 3C–F) and this explanation of regenerative ability cannot apply to the skin which is completely removed during wounding. So it is possible that *A*. *cahirinus* regenerates faster than *M*. *musculus* because of the lack of induction of inflammation and fibrosis pathways and in support of this we found that *A*. *cahirinus* expressed higher levels of *Cxcl12*, a cytokine with a known anti-inflammatory role^33^ and a role in positively regulating muscle regeneration^34,35^. Identifying cytokines such as this may well provide the molecular explanation for the improved muscle regenerative capacities of *A*. *cahirinus* and another such cytokine we identified at higher levels in *A*. *cahirinus* was adiponectin. It will be important to determine the relative levels of further cytokines such as these during muscle regeneration.

Another difference identified between *M*. *musculus* and *A*. *cahirinus* regenerating muscle was in the macrophage profiles. It is suggested that classically activated, pro-inflammatory macrophages (M1) are involved in the clearance of necrotic debris and alternatively activated anti-inflammatory or pro-regenerative macrophages (M2) provide growth factors to stimulate myofibre growth. These may appear sequentially^7^ or at the same time^26^, but whichever the case they likely perform different functions. F4/80 is a M1 marker in *A*. *cahirinus*^36^ and we failed to detect significant numbers of this macrophage subtype during *A*. *cahirinus* muscle regeneration, as was the case in skin regeneration^30^ and ear punch studies^36^ so this seems a consistent finding. This is not due to the lack of antibody reactivity because we can readily detect F4/80 macrophages in the spleen by immunocytochemistry with the same antibody^30^, markers of classical activation (*Ifn-*γ and *iNos*) can be induced in *A*. *cahirinus* spleen cells^30^ and M1 markers can be induced in *A*. *cahirinus* bone marrow derived macrophages by IFNγ and LPS^36^. On the other hand the M2 marker CD206 was present in both species during muscle regeneration with similar numbers peaking on day 4 of regeneration. The fact that M1 markers can be induced in *A*. *cahirinus* macrophages, but that they do not appear in wounds suggests that the cytokine environment must be very different between *A*. *cahirinus* and *M*. *musculus* and may play an important role in the rate or eventual outcome of the regenerative process. As a whole, however, macrophages play a critical role because in their absence regeneration is inhibited^36^ and identifying the cytokine environment and precise macrophage contribution will be an important avenue for further studies in regeneration.

Although there were no differences in the numbers of FAPS during regeneration between the two species, huge differences were seen in one of their final differentiated products, the adipocyte, after chronic muscle regeneration. These cells which are driven by IL-4/IL-13 from eosinophils to proliferate and provide beneficial growth factors for myofibre redifferentiation^26^ can differentiate into either fibroblasts or adipocytes and high levels of IL-4 inhibit adipogenic differentiation. This is the major difference we observed between *M*. *musculus* and *A*. *cahirinus* muscle regeneration – after repeated rounds of regeneration *M*. *musculus* TAs showed a large amount of adipogenic differentiation whereas *A*. *cahirinus* retained its perfect regenerative ability. It is possible that levels of IL-4 or numbers of eosinophils decline in *M*. *musculus* after repeated regeneration whereas they are maintained in *A*. *cahirinus* and it will be interesting to examine this in future experiments. In support of the idea that increased levels of cytokines could explain the improved regenerative ability of *A*. *cahirinus* (see above for Cxcl12) we found that *Adipoq* expression was upregulated to greater degree in *A*. *cahirinus* tissue after multiple rounds of regeneration. *Adipoq* is an adipokine previously thought to be expressed only by adipocytes, but has recently been shown to be expressed in several tissue types including skeletal muscle tissue. It positively regulates muscle regeneration by inducing the activation of satellite cells and by attracting non-resident myogenic cells to the injury site^35,46^. Interestingly, the chronic muscle regeneration phenotype of *M*. *musculus* with extensive adipocyte infiltration that we see here strikingly resembles that of Duchenne muscular dystrophy^27^ so understanding the molecular basis of the resistance to adipocyte infiltration in *A*. *cahirinus* may make a contribution to the understanding of this debilitating disease.

## Materials and Methods

### Animals

*A*. *cahirinus* were obtained from our breeding colony housed at University of Florida. Groups of animals, n = 3 for each sample time, of mixed sex were used, aged from 6 months to one year old. C57Bl6 *M*. *musculus* were purchased from Charles River and similarly sized mixed sex groups of animals used for the experiments of age 3 months to 6 months old. Since the gestation period of *A*. *cahirinus* is more than twice that of *Mus*, they become sexually mature at later times and the lifespan of *A*. *cahirinus* is at least twice that of *M*. *musculus* so it is problematical to know exactly what are comparable chronological ages for comparative experiments. In general the *A*. *cahirinus* animals were always chronologically older that those of *Mus*. All animals were housed and maintained by Animal Care Services at the University of Florida and all experiments were performed following the guidelines and regulations as defined in the *Guide for the Care and Use of Laboratory Animals of the National Institutes of Health*.

### Experimental procedures

All procedures involving experimental animals were approved by the Institutional Animal Care and Use Committee at the University of Florida (protocol numbers 201207707 for *A*. *cahirinus* studies and 201203505 for *M*. *musculus* studies). For tibialis anterior (TA) myotoxin experiments animals were anaesthetized with isoflurane, the hindlegs shaved and a small incision made in the skin over the superior surface of both lower hindlimbs to identify the body of the TA. The right limb TA of each animal was injected with 30 μL of a 50 μM *Naja pallida* cardiotoxin solution (CTX) (Latoxan, Valence France) and the left limb TA was injected with 30 μL of sterile PBS. At regular intervals after myotoxin injection the animals were sacrificed and the TAs removed for fixation in 4% PFA or samples were immediately frozen for immunocytochemistry or samples placed in RNAlater for qPCR.

### Histology and immunocytochemistry

The same reagents and antibodies were used on sections of both *A*. *cahirinus* and *M*. *musculus* tissues. Paraffin wax sections (10 μm), fixed frozen sections (20 μm) and unfixed frozen sections (20 μm) were all prepared by standard procedures. Wax section were used for hematoxylin and eosin staining and for Mason’s trichrome staining. Frozen sections were used for immunocytochemistry with antibodies to collagen I (Abcam ab34710) 1:100, collagen IV (Abcam ab6586) 1:100, collagen VI (Abcam ab182744) 1:100, collagen XII (Santa Cruz sc-68862) 1:100, collagen XVII (Abcam ab79878) 1:100, F4/80 (Abcam ab6640) 1:100, PDGFRα (R & D systems AF1062) 1:100, CD206 (Abcam ab64693) 1:100, perilipin (Abcam ab3526) 1:100, fibronectin (Abcam ab2413), tenascin C (Abcam ab3870) 1:100, laminin (DSHB LAM-B) 1:10, 1:100, MYHCI (DSHB A4.840) 1:1, MYHCIIA (DSHB A4.74) 1:1, MYHCIIB (DSHB BF.F3) 1:1, MYH3 (Santa Cruz sc-53091) 1:200, dystrophin (Abcam ab15277) 1:200, NF-kB (Santa Cruz sc-372) 1:200. After blocking in a mixture of 10% donkey serum, 0.5% Triton, 0.1% Tween, 0.1% fish skin gelatin for 1 hour the antibodies were diluted at the required concentration in blocking solution and incubated overnight at 4 °C. After washing the slides were incubated at room temperature in the dark for 1 hr with the relevant Alexa Fluor 488, 594 or 555 secondary antibody. Acid phosphatase staining was analysed for compromised muscle fibre integrity by incubating TA cryosections in acid phosphatase buffer (HPR reagent, 0.1 M acetate buffer pH 5.0, 50 mg/mL naphthol AS-BI phosphate) for 90 minutes at 37 °C, before being washed and counterstained with a 1:30 dilution of Harris hematoxylin for 1 minute. The stained slides were mounted using Hydromount. Succinate dehydrogenase (SDH) staining was carried out by incubating transverse muscle sections for 3 minutes at room temperature in a sodium phosphate buffer containing 75 mM sodium succinate (Sigma), 1.1 mM Nitroblue Tetrazolium (Sigma) and 1.03 mM Phenazine Methosulphate (Sigma). Samples were then fixed in 10% formal-calcium and cleared in xylene prior to mounting with DPX mounting medium (Fisher). Densitometry of the samples was performed on a Zeiss Axioskop2 microscope mounted with an Axiocam HRc camera. Axiovision Rel. 4.8 software was used to capture the images.

### RNA extraction

Tibialis anterior samples were stored in RNAlater at −80 °C until required. After thawing they were washed in nuclease-free water to remove RNAlater crystals. Tissue was homogenized using a 1600 MiniG® Tissue Homogenizer and Cell Lyser (SPEXSamplePrep) with 5/32″ stainless-steel grinding balls in TRIzol® (Ambion™) in 2 ml screw-cap vials at 1500 RPM for 3 minutes. After homogenization, the TRIZol® protocol was followed with modifications. Briefly, samples were transferred to Phase Lock Gel™ Heavy 2 mL tubes (QuantaBio). After the aqueous phase was removed to a new tube, 1 volume of 70% ethanol was added and samples mixed. At this point samples were processed according to the RNeasy Mini Kit (Qiagen 74104) or the PureLink™ RNA Mini Kit (Invitrogen 12183018 A) according to the manufacturers’ protocols, starting from the addition of sample to kit column tubes. After elution, samples were quantified and RNA integrity number determined using a 4200 TapeStation (Agilent) (all RINs > 6 for RT-PCR).

### RT-qPCR Analysis

cDNA was generated from 1.5 μg of RNA using SuperScript ™ IV VILO Reverse Transcriptase (Invitrogen 11756050) following the manufacturers protocol. Real-Time PCR was performed using Sso-Fast™ EvaGreen® Supermix (Bio-Rad 172–5200) on a Bio-Rad C1000 Touch™ Thermal Cycler. Fold change in expression was calculated using the ΔΔCt relative expression method^47^. *A*. *cahirinus-*specific and *M*. *musculus-*specific primers were used for qPCR to avoid erroneous readings due to sequence differences (see Supplementary Table 1 for details). *A*. *cahirinus* sequences were generated from our skin transcriptome data.

### Biomechanical properties of the TA

Ten unfixed TA samples were characterized using a custom indentation device. TA was excised immediately after each animal was euthanized. Six distinct locations were indented per muscle, and all indentations were completed within 10 mins of excision. A 4-mm spherical probe was used to perform 200 µm indents (<10% of sample thickness), and Steady-State Modulus (SSM) values were calculated as described elsewhere^48,49^. Briefly, samples were indented and allowed to undergo stress relaxation for 20 seconds before probe retraction. SSM describes the effective stiffness of the muscle after indentation is held. Muscles were submerged in culture medium to prevent dehydration, reduce cell death, and minimize adhesion effects during indentation.

### Statistical analysis

Data are presented as mean± SE. Significant differences among groups were analyzed by wither 2-tailed T-test or one-way analysis of variance (ANOVA) followed by Bonferroni correction for multiple comparison tests as appropriate. Statistical analysis was performed on GraphPad Prism software. Differences were considered statistically significant at ^*^p < 0.05, ^**^p < 0.01 or ^***^p < 0.001.

### Data availability statement

After publication the materials, data and associated protocols contained in the manuscript will be made freely available.

## Electronic supplementary material

Supplementary data

## References

[1] Charge SBP, Rudnicki MA (2004). Cellular and molecular regulation of muscle regeneration. Physiol. Rev..

[2] Musaro, A. The basis of muscle regeneration. *Advances in Biology*, 10.1155/2014/612471 (2014).

[3] Arnold L (2007). Inflammatory monocytes recruited after skeletal muscle injury switch into anitinflammatory macrophages to support myogenesis. JEM.

[4] Saclier M, Cuvellier S, Magnan M, Mounier R, Chazaud B (2013). Monocyte/macrophage interactions with myogenic precursor cells during skeletal muscle regeneration. FEBS J..

[5] Aurora AB, Olson EN (2014). Immune modulation of stem cells and regeneration. Cell Stem Cell.

[6] Martinez CO (2010). Regulation of skeletal muscle regeneration by CCR2-activating chemokines is directly related to macrophage recruitment. Am. J. Physiol. Regul. Integr. Comp. Physiol..

[7] Sunman M (2006). Macrophages and skeletal muscle regeneration: a clodronate-containing liposome depletion study. Am. J. Physiol. Regul. Integr. Comp. Physiol..

[8] Sun D (2009). Bone marrow-derived cell regulation of skeletal muscle regeneration. Monocyte/macrophage interactions with myogenic precursor cells during skeletal muscle regeneration. FASEB J..

[9] Tidball JG, Wehling-Henricks M (2007). Macrophages promote muscle membrane repair and muscle fibre growth and regeneration during modified muscle loading in mice *in vivo*. J. Physiol..

[10] Lepper C, Partridge TA, Fan CM (2014). An absolute requirement for Pax7-positive satellite cells in acute injury-induced skeletal muscle regeneration. Development..

[11] Sambasivan. R (2011). Pax7-expressing satellite cells are indispensable for adult skeletal muscle regeneration. Development.

[12] Yan Z (2003). Highly coordinated gene regulation in mouse skeletal muscle regeneration. J. Biol. Chem..

[13] Conboy IM, Rando TA (2002). The Regulation of Notch Signaling Controls Satellite Cell Activation and Cell Fate Determination in Postnatal Myogenesis. Developmental Cell.

[14] Brack AS, Conboy IM, Conboy MJ, Shen J, Rando TA (2008). A temporal switch from Notch to Wnt signaling in muscle stem cells is necessary for normal adult myogenesis. Cell Stem Cell.

[15] Murphy MM (2014). Transiently active Wnt/beta-catenin signaling is not required but must be silenced for stem cell function during muscle regeneration. Stem Cell Reports.

[16] Chakkalakal JV, Jones KM, Basson MA, Brack AS (2012). The aged niche disrupts muscle stem cell quiescence. Nature.

[17] Conboy IM (2005). Rejuvenation of aged progenitor cells by exposure to a young systemic environment. Nature.

[18] Egerman MA (2015). GDF11 Increases with age and inhibits skeletal muscle regeneration. Cell Metab.

[19] Bentzinger CF (2013). Fibronectin regulates Wnt7a signaling and satellite cell expansion. Cell Stem Cell..

[20] Tierney MT (2014). Autonomous Extracellular Matrix Remodeling Controls a Progressive Adaptation in Muscle Stem Cell Regenerative Capacity during Development. Cell Rep.

[21] Lukjanenko L (2016). Loss of fibronectin from the aged stem cell niche affects the regenerative capacity of skeletal muscle in mice. Nat Med..

[22] Urciuolo A (2013). Collagen VI regulates satellite cell self-renewal and muscle regeneration. Nat Commun..

[23] Engler AJ, Sen S, Sweeney HL, Disher DE (2006). Matrix elasticity directs stem cell lineage specification. Cell.

[24] Joe AWB (2010). Muscle injury activates resident fibro/adipogenic progenitors that facilitate myogenesis. Nat Cell Biol.

[25] Uezumi A, Fukada S, Yamamoto N, Takeda S, Tsuchida K (2010). Mesenchymal progenitors distinct from satellite cells contribute to ectopic fat cell formation in skeletal muscle. Nat Cell Biol.

[26] Heredia JE (2013). Type 2 innate signals stimulate fibro/adipogenic progenitors to facilitate muscle regeneration. Cell.

[27] Banker, B. Q. & Engel, A. G. Basic reactions of muscle. In *Myology*, Vol 1 (eds A. G. Engel & C. Franzini-Armstrong) 691–747 (McGraw-Hill, New York, 2004).

[28] Seifert AW (2012). Skin shedding and tissue regeneration in African spiny mice (A. cahirinus). Nature.

[29] Brant JO, Lopez M-C, Baker HV, Barbazuk WB, Maden M (2015). A comparative analysis of gene expression profiles during skin regeneration in M. musculusand A. cahirinus. PlosOne.

[30] Brant JO, Yoon JH, Polvadore T, Barbazuk B, Maden M (2016). Cellular events during scar-free healing in the spiny mouse, A. cahirinus. Wound Rep Regen.

[31] Gawriluk TR (2016). Comparative analysis of ear-hole closure identifies epimorphic regeneration as a discrete trait in mammals. Nat Commun..

[32] Hynes RO (2009). The extracellular matrix: not just pretty fibrils. Science.

[33] McCandless EE (2006). CXCL12 limits inflammation by localizing mononuclear infiltrates to the perivascular space during experimental autoimmune encephalomyelitis. J Immunol.

[34] Brzoska E (2012). Sdf-1 (CXCL12) improves skeletal muscle regeneration via the mobilisation of Cxcr4 and CD34 expressing cells. Biol Cell.

[35] Maeda Y (2017). CXCL12 and osteopontin from bone marrow-derived mesenchymal stromal cells improve muscle regeneration. Sci Rep.

[36] Simkin J, Gawriluk TR, Gensel JC, Seifert AW (2017). Macrophages are necessary for epimorphic regeneration in African spiny mice. eLife.

[37] Fiaschi T (2014). Adiponectin as a tissue regenerating hormone: more than a metabolic function. Cell Mol Life Sci.

[38] Gawtor, M. & Proszynski, T. J. The molecular cross talk of the dystrophin-glycoprotein complex. *Ann NY Acad Sci* 1–11, 10.1111/nyas.13500 (2017). 10.1111/nyas.13500 29068540

[39] Engler AJ (2004). Myotubes differentiate optimally on substrates with tissue-like stiffness: pathological implications for soft or stiff microenvironments. J Cell Biol.

[40] Alfuraih AM (2018). The effect of unit, depth, and probe load on the reliability of muscle shear wave elastography: Variables affecting reliability of SWE. J Clin Ultrasound.

[41] Yin, L., et al.. Three-dimensional shear wave elastography of skeletal muscle: preliminary study. *J Ultrasound Med*10.1002/jum.14559 (2018). 10.1002/jum.14559 29399850

[42] Le Sant G (2017). Stiffness mapping of lower leg muscles during passive dorsiflexion. J Anat.

[43] Wijesinghe P (2017). Ultrahigh-resolution optical coherence elastography images cellular-scale stiffness of mouse aorta. Biophys J.

[44] Swift, J. *et al*. Nuclear lamin-A scales with tissue stiffness and enhances matrix-directed differentiation. *Science* **341**, 10.1126/science.1240104 (2013). 10.1126/science.1240104 PMC3976548 23990565

[45] Smith LR, Barton ER (2014). Collagen content does not alter the passive mechanical properties of fibrotic skeletal muscle in mdx mice. Am. J. Physiol.-Cell Physiol..

[46] Fiaschi T (2012). Globular adiponectin activates motility and regenerative traits of muscle satellite cells. PLoS ONE.

[47] Livak KJ, Schmittgen TD (2001). Analysis of relative gene expression data using real-time quantitative PCR and the 2(-Delta Delta C(T)) method. Methods.

[48] Stewart DC, Rubiano A, Dyson K, Simmons CS (2017). Mechanical characterization of human brain tumors from patients and comparison to potential surgical phantoms. PLoS ONE.

[49] Rubiano A (2016). Stem cell therapy restores viscoelastic properties of myocardium in rat model of hypertension. J Mech Behav Biomed Mater.

