# Supplementary material for: Perfect chronic skeletal muscle regeneration in adult spiny mice, Acomys cahirinus
Source: Sci Rep. 2018 Jun 11;8:8920. doi: 10.1038/s41598-018-27178-7 (PMC5995887; doi:10.1038/s41598-018-27178-7)
Supplement: Supplementary file 1 — Supplementary data [file 41598_2018_27178_MOESM1_ESM.pdf]

SUPPLEMENTARY INFORMATION

MANUSCRIPT TITLE: **Perfect chronic skeletal muscle regeneration in adult spiny mice, *Acomys cahirinus*.**

AUTHORS: Malcolm Maden, Jason Orr Brant, Andres Rubiano, Aaron Gabriel W. Sandoval, Chelsey Simmons, Robert Mitchell, Henry Collin-Hooper, Jason Jacobson, Saleh Omairi, Ketan Patel

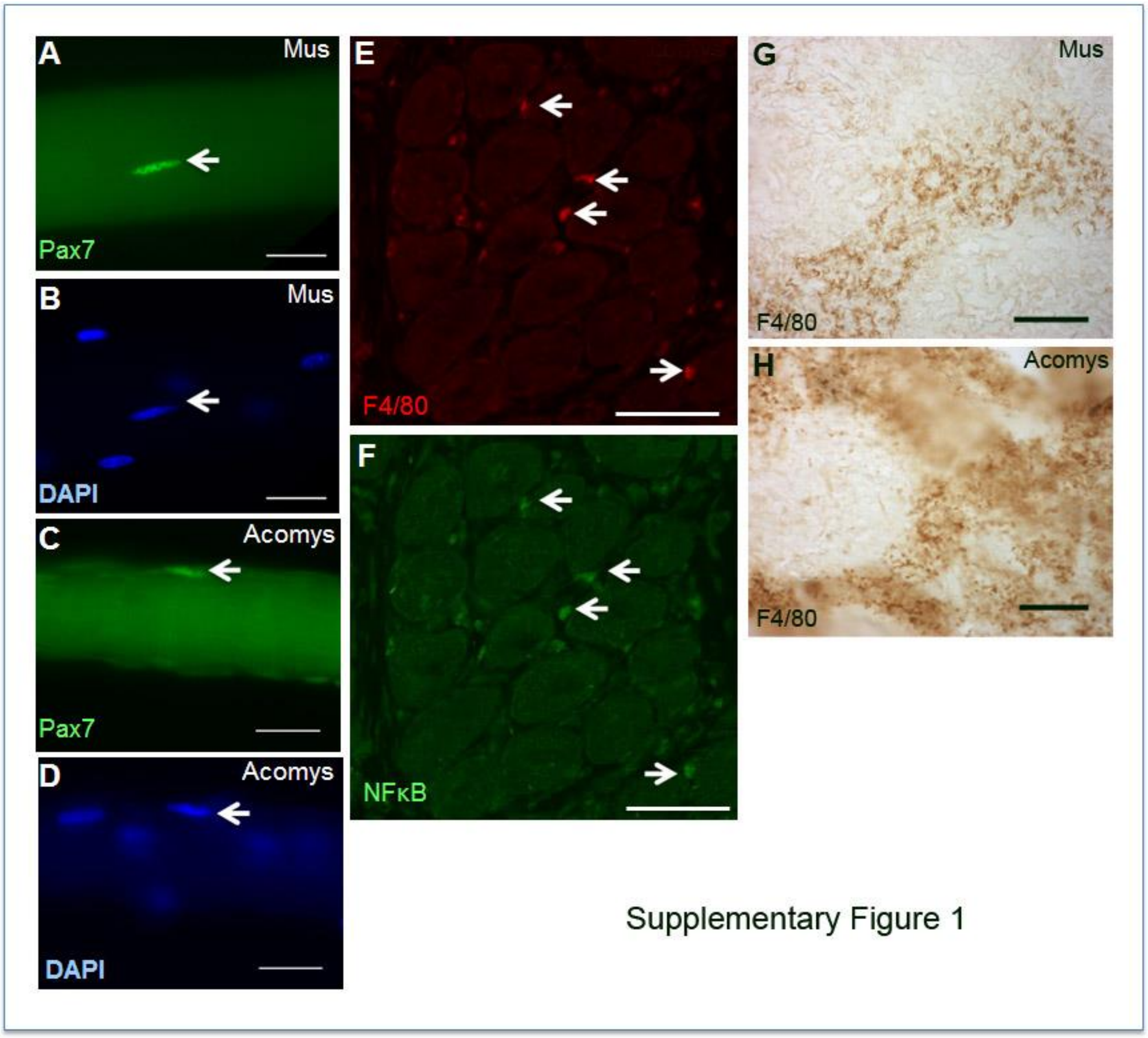

<u>Supplementary Fig. 1</u>. **A-D** Single fibre immunostaining for expression of satellite cells marker Pax7 on freshly isolated *M. musculus* (A, B) and *A. cahirinus* (C, D) muscle fibres. Arrows show expression of Pax7 co-localizing to a DAPI stained nuclei. Scale bars = 100μm. **E-F**. Co-localisation of macrophage marker F4/80 to the expression of NFκB (indicated by arrows). Scale bars = 200μm. G-H, Immunolocalisation of F4/80 antibody in *M. musculus* (**G**) and *A. cahirinus* (**H**) spleen showing +ve reaction around the follicles. Scale bars = 100μm.

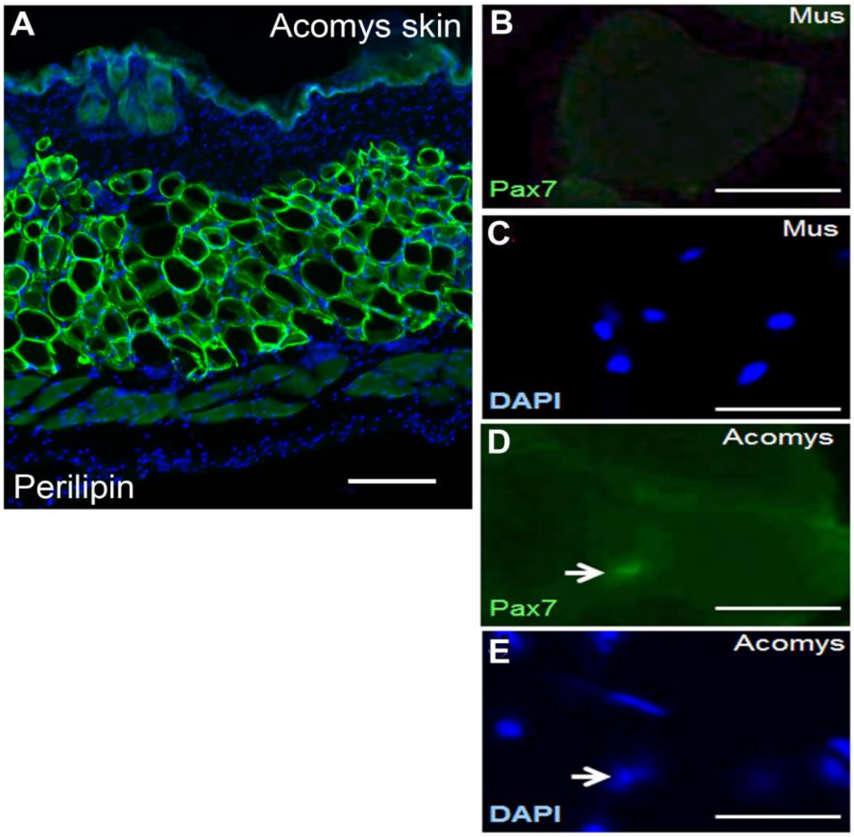

Supplementary Fig. 2. **A** Perilipin immunofluorescence on *A. cahirinus* normal skin showing adipocytes in the hypodermis. Scale bar = 200µm. **B - C** lack of expression of Pax7 in *M. musculus* muscle after five rounds of CTX damage. **D - E** Expression of Pax7 in *A. cahirinus* muscle after five rounds of CTX damage. Scale bars = 100µm.

Acomys RT Primers

Gene	Forward Primer Sequence	Reverse Primer Sequence
Myog	CTTTTCTTCCAGGGGACCTC	ACCAGGAGCCCCACTTCTAT
Myf5	CAGCTCAGCTTTGTGTGCTC	GCTGCCAGTTCTCACCTTCT
MyoD1	TCTGGCCAAGCGACTCTTAT	TCCAGTCCTGCGTGTAACAG
Myh3	ATGTCGATGGAAGCCAAGAG	GGTTCCCCAACAGGATTCTC
Col1a1	GGCTCCTCGTTTTCCTTCTT	CCCTGGAAACAAAGGAGACA
Col3a1	GACCTCGTGCTCCAGTAAGC	CTGGACCAAAAGGTGATGCT
Col12a1	GTGCGCAAAACATCTCAGAA	CGTACAATGGGCAAGGCTAT
Tgf-B1	GAGCGCACGATCATGTTG	CTGCCCCTACATCTGGAGTC
NF-KB	CTGGGCCAGAATTGGATAAA	AGGGACTCGTGCATGTTCTC
Cxcl12	TGGGCTGTTGTGCTTACTTG	CCAAACTGTGCCCTTCAGAT
Adipoq	TTAAGGGACCTCTGCCAAGA	CTGGAGTCACGCCTGTGTTA
Gapdh	CGACCTTCACCATCTTGTCA	CCCACCAACCTGGTTCCTAT

Mus RT Primers

Gene	Forward Primer Sequence	Reverse Primer Sequence
Myog	ACCAGGAGCCCCACTTCTAT	GTCCCCAGTCCCTTTTCTTC
Myf5	CAGCTCAGCTTTGTGTGCTC	GCCAGTTCTCCCCTTCTGAG
MyoD1	GCCACTCAGGTCTCAGGTGT	GCTCTGGCCAAGCAACTCT
Myh3	ATGAGTAGCGACACCGAGATG	ACAAAGCAGTAGGTTTTGGCAT
Col1a1	CTCCTGGCAACAAAGGAGAC	GAAGGTCCAGGCTCACCAC
Col3a1	CCTGGCTCAAATGGCTCAC	GACCTCGTGTTCCGGGTAT
Col12a1	TGCACCTGAGCCATAGAGTG	ATTCAAGGCCTTCCGAATTT
Tgf-B1	TGCCCTCTACAACCAACACA	GTTGGACAACTGCTCCACCT
NF-KB	TGCACCTGAGCCATAGAGTG	ATTCAAGGCCTTCCGAATTT
Cxcl12	TGCATCAGTGACGGTAAACCA	CACAGTTTGGAGTGTTGAGGAT
Adipoq	TGTTCCTCTTAATCCTGCCCA	CCAACCTGCACAAGTTCCCTT
Gapdh	TGACCTCAACTACATGGTCTACA	CTTCCCATTCTCGGCCTTG

Supplementary Table 1. Primer sequences used for qPCR analyses.
